# Speech‐In‐Noise Perception in Alzheimer's Disease and Primary Progressive Aphasia

**DOI:** 10.1111/ene.70701

**Published:** 2026-07-14

**Authors:** Sophie A. Froud, Benjamin A. Levett, Jessica Jiang, Lucy B. Core, Nehzat Koohi, Madison Tutton, Tim Green, Doris‐Eva Bamiou, Charles R. Marshall, Chris J. D. Hardy, Jason D. Warren

**Affiliations:** ^1^ Dementia Research Centre, Queen Square Institute of Neurology University College London London UK; ^2^ Department of Clinical and Movement Neurosciences, Institute of Neurology University College London London UK; ^3^ The Ear Institute University College London London UK; ^4^ Eargym Ltd London UK; ^5^ Speech, Hearing and Phonetic Sciences, Chandler House University College London London UK; ^6^ National Hospital for Neurology and Neurosurgery, Queen Square London UK; ^7^ National Institute for Health and Care Research, UCLH/UCL Biomedical Research Centre (Hearing Theme) London UK; ^8^ Centre for Preventive Neurology Queen Mary University of London London UK

**Keywords:** Alzheimer's disease, auditory perception, frontotemporal dementia, primary progressive aphasia, speech perception

## Abstract

**Background:**

Understanding speech despite background noise is essential for everyday communication, but makes heavy neural processing demands. It is therefore potentially vulnerable to neurodegenerative diseases, particularly those led by communication deficits (primary progressive aphasia). However, how speech‐in‐noise perception is affected in these diseases is poorly understood.

**Methods:**

Here we addressed this in 59 patients representing typical Alzheimer's disease and canonical logopenic, nonfluent/agrammatic and semantic variant syndromes of primary progressive aphasia, compared with 24 cognitively‐well, older controls. We administered a digit triplet test of speech‐in‐noise perception based on the task used in the UK Biobank study, alongside pure tone audiometry and a general neuropsychological assessment. Voxel‐based morphometry of patients' brain MRI scans was used to identify structural neuroanatomical associations of speech‐in‐noise perception.

**Results:**

After adjusting for age, peripheral hearing and general cognitive function, the Alzheimer's, logopenic and nonfluent primary progressive aphasia groups performed significantly worse on speech‐in‐noise perception than controls. The nonfluent primary progressive aphasia and Alzheimer groups additionally had significantly worse peripheral hearing function than controls. Speech‐in‐noise perceptual performance correlated with grey matter atrophy in the right supramarginal gyrus.

**Conclusion:**

Profiles of central (brain) and peripheral hearing impairment stratify major dementias, with implications for diagnosis and development of interventions to improve real‐world communication in people living with dementia.

## Graphical abstract

Speech‐in‐noise perception is impaired in Alzheimer's disease and primary progressive aphasia when accounting for peripheral hearing and general cognitive functions, and is associated with grey matter volume in the right supramarginal gyrus.

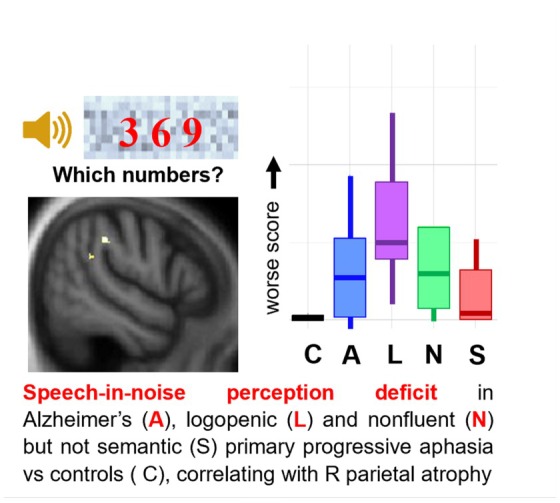

## 1 Introduction

Hearing impairment is linked to dementia [1], however the nature of this linkage is not well understood [2]. Neurodegenerative diseases target the auditory brain [3, 4], and pure tone audiometry (PTA)—a standard measure of peripheral hearing function—poorly predicts real‐world hearing disability in people with dementia [5]. Successful communication in daily life typically depends on understanding spoken messages and paralinguistic auditory cues under changing, noisy listening conditions that make substantial computational demands on auditory cortical mechanisms: these ‘brain hearing’ mechanisms are vulnerable in Alzheimer's disease (AD) and other dementias [4, 6]. While impairment of episodic memory is the most widely recognised clinical feature [7], increased listening difficulty in noisy environments due to impaired auditory scene analysis (the auditory cognitive operation whereby sound objects of interest such as phonemes are parsed from the acoustic background) is also a common early feature of ad [4, 8]. The ‘language‐led’ dementias or primary progressive aphasias (PPA) are generally characterised clinically in terms of speech and language deficits but also have distinctive auditory phenotypes, with sometimes marked changes in hearing from an early stage of the illness [9, 10]. The language variant of AD (logopenic variant PPA, lvPPA) is associated with increased difficulty listening in background noise analogous to typical AD [10], compounded by impaired phonological working memory [4]; while the nonfluent‐agrammatic variant of PPA (nfvPPA) variably affects pure tone detection [11] and perception of auditory patterns underpinning vocal identity and accents [12, 13] as well as auditory scene analysis [6]. The semantic variant of PPA (svPPA) has a core deficit of semantic memory affecting recognition of sensory objects including voices and nonverbal sounds [13, 14]; auditory perceptual processing in this syndrome is modulated by semantic predictability [15, 16]. These auditory phenotypes are grounded in distinct patterns of neural network dysfunction and atrophy affecting temporo‐parietal cortices in AD and lvPPA [8, 17, 18], peri‐sylvian atrophy in nfvPPA [19] and the anterior temporal lobes in svPPA [13, 14].

While both brain hearing (auditory cognitive) deficits and peripheral hearing loss are likely to contribute to hearing impairment associated with neurodegenerative pathologies, their relative contributions have not been defined. Besides potential utility in early dementia detection, auditory brain dysfunction in these diseases is likely to demand hearing management strategies beyond the audiometric focus of conventional hearing aids. Speech‐in‐noise perception is a widely tested aspect of hearing that is key to everyday listening and likely to engage auditory brain mechanisms [20, 21]. As AD and PPA syndromes impact related aspects of auditory scene analysis including sound source segregation, dichotic listening and the ‘cocktail party effect’ [6, 22, 23], speech‐in‐noise processing is likely to be vulnerable in these diseases. Indeed, hearing impairment measured using a test of speech‐in‐noise perception was associated with increased risk of incident dementia over 11 years of follow‐up in the UK Biobank cohort [24]. However, as an index of hearing function in dementia, speech‐in‐noise perception has been much less widely studied than PTA. This may signify an under‐appreciation of the role of brain hearing (and longstanding emphasis on memory and language deficits) in major dementia syndromes, as well as the multi‐componential nature of speech‐in‐noise processing, which engages general cognitive capacities (such as working memory and executive function) and peripheral hearing function in addition to auditory scene analysis per se [20]. Clarifying the status of speech‐in‐noise perception in these diseases, particularly in relation to PTA, may help to further define auditory cognitive phenotypes in AD and PPA syndromes, with implications for diagnosis and development of interventions to improve real‐world communication in people living with dementia.

Here we compared speech‐in‐noise perception and PTA in a cohort of patients representing typical AD and all major syndromes of PPA versus cognitively‐healthy older individuals. We administered the digit triplet test as a standard, widely‐used tool for assessing speech‐in‐noise perception [25], and assessed its relation both to daily‐life hearing symptoms and to structural neuroanatomical correlates of hearing test performance using voxel‐based morphometry of patients' brain MRI scans. In line with previous work on auditory cognition in these diseases [4, 6, 8, 9, 10, 11, 12, 13, 14, 15, 16, 19, 20, 21], we hypothesised that speech‐in‐noise perception would be impaired in typical AD, lvPPA and nfvPPA, after accounting for peripheral hearing function (PTA performance) and general cognitive abilities, due to specific deficits of auditory cognition (auditory scene analysis); whereas in svPPA, speech‐in‐noise perception would be relatively intact, given the very constrained semantic predictability of digit names. We further hypothesised that impaired speech‐in‐noise perception (but not PTA performance) would have a neuroanatomical correlate within the temporo‐parietal neural network involved in these syndromes, and previously implicated in the processing of acoustically degraded speech in AD and PPA syndromes and in the healthy brain [6, 14, 17, 19, 20, 22, 23].

## 2 Materials and Methods

### 2.1 Participants

Fifty‐nine patients (23 with typical memory‐led AD, 12 with lvPPA, 8 with nfvPPA, 16 with svPPA) and 24 cognitively‐well older individuals with no history of neurological or psychiatric disorders participated. All patients had clinically mild‐to‐moderate disease [26, 27] and fulfilled consensus clinical diagnostic criteria with supportive volumetric brain MRI and behavioural phenotype. CSF or plasma AD biomarkers were positive based on local reference ranges for all patients with a relevant clinical diagnosis who underwent testing (nine AD, five lvPPA). Participants with a maximum forward digit span score > 3 were included to ensure they were able to repeat the three digits needed for the speech‐in‐noise task. No participant had a history of otological disease other than presbycusis (six patients: one AD, one nfvPPA, four svPPA); hearing aid users were allowed to wear their aids during testing. All participants underwent a general neuropsychological assessment (Table 1) to corroborate the syndromic diagnosis and provide measures of potentially confounding cognitive processes for incorporation in the regression analyses.

**TABLE 1:** General demographic, clinical, and neuropsychological characteristics of participant groups.

Characteristic	Controls	AD	lvPPA	nfvPPA	svPPA	Statistical test
Demographic and clinical						
Male:Female	6:18	**9:14**	**10:2**	**5:3**	**12:4**	*p* = 0.016
Age (years)	68.2 (6.6)	**73.9 (7.9)** ^a^	68.6 (6.4)	**72.9 (6.6)** ^a^	**65.3 (8.6)**	*F*(4,78) = 3.923, *p* = 0.006
Handedness L:R	1:21	1:13	2:9	0:7	**6:6**	*p* = 0.017
Education (years)	16.5 (2.8)^2^	16.2 (2.7)^1^	15.0 (2.3)	14.0 (2.0)^1^	15.6 (3.3)^2^	*X* ^2^ (4) = 7.507, *p* = 0.111
Symptoms (years)	NA	4.9 (3.3)^1^	4.4 (2.4)	2.8 (0.8)^2^	3.9 (1.7)^2^	*X* ^2^ (3) = 2.420, *p* = 0.490
MMSE (/30)	28.9 (1.2)	**20.9 (5.2)**	**18.4 (5.9)** ^c^	**25.8 (2.6)**	**22.1 (8.65)**	*X* ^2^ (4) = 42.324, *p* < 0.001
Neuropsychological functions						
Executive function						
WASIMR (/32)	26.1 (2.4)	**17.0 (7.2)** ^**1**,a^	**12.8 (7.7)** ^a^	**12.4 (9.0)** ^a^	24.9 (7.9)	*X* ^2^ (4) = 38.826, *p* < 0.001
Verbal fluency: letter (total)	17.1 (3.5)^15^	12.5 (8.4)^12^	9.0 (7.3)^4^	5.0 (6.0)^3^	**8.9 (5.4)** ^**4**^	*X* ^2^ (4) = 12.600, *p* = 0.013
Category (total)	24.9 (3.5)^15^	**11.2 (6.8)** ^**12**^	**7.0 (4.5)** ^**4**^	**11.7 (5.3)** ^**1**^	**10.6 (6.6)** ^**4**^	*X* ^2^ (4) = 21.596, *p* < 0.001
Elevator attention: Counting (/6)	6.0 (0)^1^	**5.1 (1.8)** ^**4**^	5.2 (2.0)^3^	**5.7 (0.5)** ^**2**^	5.8 (0.6)^2^	*X* ^2^ (4) = 8.502, *p* = 0.06
Distraction (/9)	6.6 (3.1)^6^	**1.8 (2.4)** ^**13**^	**1.7 (3.6)** ^**8**^	3.7 (2.3)^3^	6.1 (4.0)^6^	*X* ^2^ (4) = 17.390, *p* = 0.002
Verbal working memory						
DS forward (max)	7.2 (0.8)	6.4 (1.1)^b^, ^c^	**4.6 (0.8)** ^a^	**4.9 (0.6)** ^a^	6.8 (0.9)	*X* ^2^ (4) = 43.657, *p* < 0.001
DS reverse (max)	5.1 (1.6)^1^	3.8 (1.0)^2^	**3.1 (0.5)** ^**1**^,^a^	5.3 (5.8)^2^	5.1 (1.3)	*X* ^2^ (4) = 21.503, *p* < 0.001
Speech processing						
PALPA‐3 (/36)	35.9 (0.27)^10^	35.0 (1.7)^20^	25.6 (17.5)^5^	35.3 (0.5)^2^	35.3 (1.0)^3^	*X* ^2^ (4) = 6.880, *p* = 0.14
Bi‐syllabic word repetition (/15)	14.8 (0.41)^13^	NA	13.2 (1.3)^8^	14.2 (1.0)^2^	14.3 (0.9)^5^	*X* ^2^ (3) = 6.700, *p* = 0.072
Semantic memory						
BPVS (/150)	146.0 (0.6)^21^	144.0 (10.3)^2^,^a^	138.0 (16.4)^1^,^a^	142.0 (6.4)^1^,^a^	**106.0 (40.0)**	*X* ^2^ (4) = 26.450, *p* < 0.001
Episodic memory						
CPAL (/24)	19.7 (2.0)^15^	**4.9 (5.4)** ^**10**^,^c^	**1.0 (1.4)** ^**4**^,^c^	17.7 (4.6)^1^	**8.1 (7.7)** ^**4**^	*X* ^2^ (4) = 29.312, *p* < 0.001
Other skills						
VOSP‐OD (/20)	18.8 (1.1)	**14.4 (5.3)** ^**1**^	**11.2 (8.4)**	17.4 (1.8)	17.2 (2.8)	*X* ^2^ (4) = 28.088, *p* < 0.001
Arithmetic (/24)	14.6 (3.9)^14^	**6.1 (5.6)** ^11^	**1.5 (2.0)** ^10^,^a^, ^c^	**6.0 (2.6)** ^**2**^	12.1 (6.3)^5^	*X* ^2^ (4) = 22.868, *p* < 0.001
Hearing						
PTABEA (dB)	15.6 (7.4)	25.7 (13.6)	20.1 (15.6)	**26.1 (8.5)**	22.7 (14.1)	*F*(4,78) = 2.465, *p* = 0.02
SRT	−11.8 (0.5)	**−10.1 (2.3)**	**−8.9 (2.0)** ^a^, ^b^, ^c^	**−10.4 (1.4)**	−11.0 (1.4)	*F*(4,78) = 7.628, *p* < 0.001
mAIAD	106 (5.2)^2^	97.2 (13.9)^4^	**91.0 (12.3)** ^**2**^	86.0 (20.2)^2^	**88.9 (12.9)** ^**3**^	*X* ^2^ (4) = 19.565, *p* < 0.001

*Note:* Mean (standard deviation) values are shown for continuous measures and counts for categorical variables; superscript numerals indicate the number of participants with missing data for that item. An omnibus test was used for all group comparisons apart from PTABEA and SRT, for which we used regression outputs. Values in bold indicate significant patient group differences compared to the cognitively‐healthy older listener control group. All significant results correspond to omnibus test *p* values < 0.05.

Abbreviations: AD, patient group with typical Alzheimer's disease; BPVS, British Picture Vocabulary Scale; CPAL, Camden Paired Associates Learning; DS, digit span; L, left; lvPPA, patient group with logopenic variant primary progressive aphasia; mAIAD, modified Amsterdam Inventory for Auditory Disability and Handicap; MMSE, Mini Mental State Examination score; NA, not available; nfvPPA, patient group with nonfluent variant primary progressive aphasia; PALPA3, Psycholinguistic Assessment of Language Processing in Aphasia subtest 3 (minimal pairs discrimination); PTABEA, pure tone audiometry better ear average; R, right; SRT, 50% speech reception threshold (see text); svPPA, patient group with semantic variant primary progressive aphasia; VOSP‐OD, Visual Object Space Perception Object Decision; WASIMR, Wechsler Adult Scale of Intelligence Matrix Reasoning.

^a^ Significant difference versus the svPPA group.

^b^ Significant difference versus the lvPPA group.

^c^ Significant difference versus the nfvPPA group.

All participants gave informed consent and ethical approval was granted by the University College London National Hospital for Neurology and Neurosurgery Joint Research Ethics Committees, following Declaration of Helsinki guidelines.

### 2.2 Hearing Assessments

All participants had PTA following a standard protocol (details in Supporting Information). Across the range of frequencies (500–4000 Hz) tested for each ear, a ‘better ear average’ (BEA) threshold was calculated for each participant.

An adapted version of the digit triplet test from the UK Biobank study [24, 25] was used to measure speech‐in‐noise perception. As auditory stimuli, spoken digits are highly familiar, phonologically simple and approximately balanced in frequency and length, allowing more precise control over stimulus properties such as phonetic composition and signal‐to‐noise ratio, while minimising potentially confounding influences from vocabulary knowledge and lexical analysis (important considerations in PPA syndromes) [15]. Sequences of three spoken digits mixed with varying levels of speech‐weighted white noise were presented diotically via headphones in a quiet room; on each trial, the participant was asked to repeat the digits heard and the speech signal‐to‐noise ratio was adjusted following an adaptive psychophysical staircase protocol, spanning a range of signal‐to‐noise characteristics typically encountered in everyday listening environments. Fifteen trials were presented. To reflect the methodology of the Biobank study, a 50% speech reception threshold (SRT) was calculated for each individual. In the present study, this SRT score was based on the proportion of digits repeated correctly across trials at each signal‐to‐noise ratio tested (details in Supporting Information).

Daily‐life hearing symptoms were indexed using study partners' (for patients) and cognitively‐healthy volunteers' responses to the Modified Amsterdam Inventory for Auditory Disability and Handicap (mAIAD) [28].

### 2.3 Behavioural Data Analyses

Data were analysed using Excel and R studio (v4.4.1). For continuous demographic and neuropsychological data, participant groups were compared using ANOVA and Kruskal–Wallis tests (dependent on normality of data); group categorical data were compared using Fisher's exact tests.

Participant groups were compared on PTABEA and SRT using regression models. Performance on the digit triplet test could potentially be confounded by various factors apart from disease effects on the brain hearing process of interest; these include age, peripheral hearing function, auditory working memory and general executive capacity. We wished to adjust for these potential confounds, as well as balancing the models as directly as possible to allow a direct comparison between speech‐in‐noise and PTA performance. Accordingly, each regression model incorporated the PTABEA or SRT test score as a dependent variable, with covariates of age, sex, forward digit span (indexing auditory verbal working memory) and the Wechsler Adult Scale of Intelligence Matrix Reasoning (WASIMR, indexing nonverbal executive function); the SRT model additionally incorporated PTABEA as a covariate. Where a significant overall main group effect was identified, post hoc pairwise comparison *t*‐tests were conducted to identify the between‐group differences driving the effect. To mitigate the concern that SRT ceiling effects may have influenced the results, a non‐parametric Kruskal–Wallis test was conducted alongside the main regression analysis, as well as a replication of the main regression excluding those participants performing at ceiling. Effect size estimates were extracted.

In order to determine whether peripheral hearing function and speech‐in‐noise perception were related, and how performance on these hearing tests was related to general demographic and executive factors and to daily‐life hearing, we assessed associations of PTABEA and SRT with each other and with auditory working memory and mAIAD scores across all groups, in the combined patient groups and within the AD group using Spearman's correlations.

For all tests, a threshold *p* < 0.05 was accepted as the criterion for statistical significance.

### 2.4 Neuroimaging Data Analysis

Volumetric brain MRI scans were acquired for 42 patients in the cohort (13 AD, 11 lvPPA, 6 nfvPPA and 12 svPPA), and were pre‐processed using SPM12 (Department of Imaging Neuroscience, https://www.fil.ion.ucl.ac.uk/spm/), following standard protocols (details in Supporting Information). In a VBM analysis over the combined patient cohort, voxel‐wise grey matter intensity was modelled as an inverse function of raw PTABEA and SRT scores, with covariates of age, total intracranial volume, diagnostic group membership, nonverbal executive functioning (WASIMR) and auditory verbal working memory (forward digit span). Statistical parametric maps were generated using an initial cluster‐defining threshold (*p* < 0.001) and assessed at a peak‐level significance threshold (*p* < 0.05), after family‐wise error (FWE) correction for multiple voxel‐wise comparisons within pre‐specified neuroanatomical regions of interest: these regions (shown in Figure S1) comprised posteromedial, lateral temporoparietal, dorsolateral prefrontal cortices and Heschl's gyrus, based on previously demonstrated neuroanatomical correlates of speech‐in‐noise perception [4, 20, 23].

## 3 Results

**FIGURE 1:**
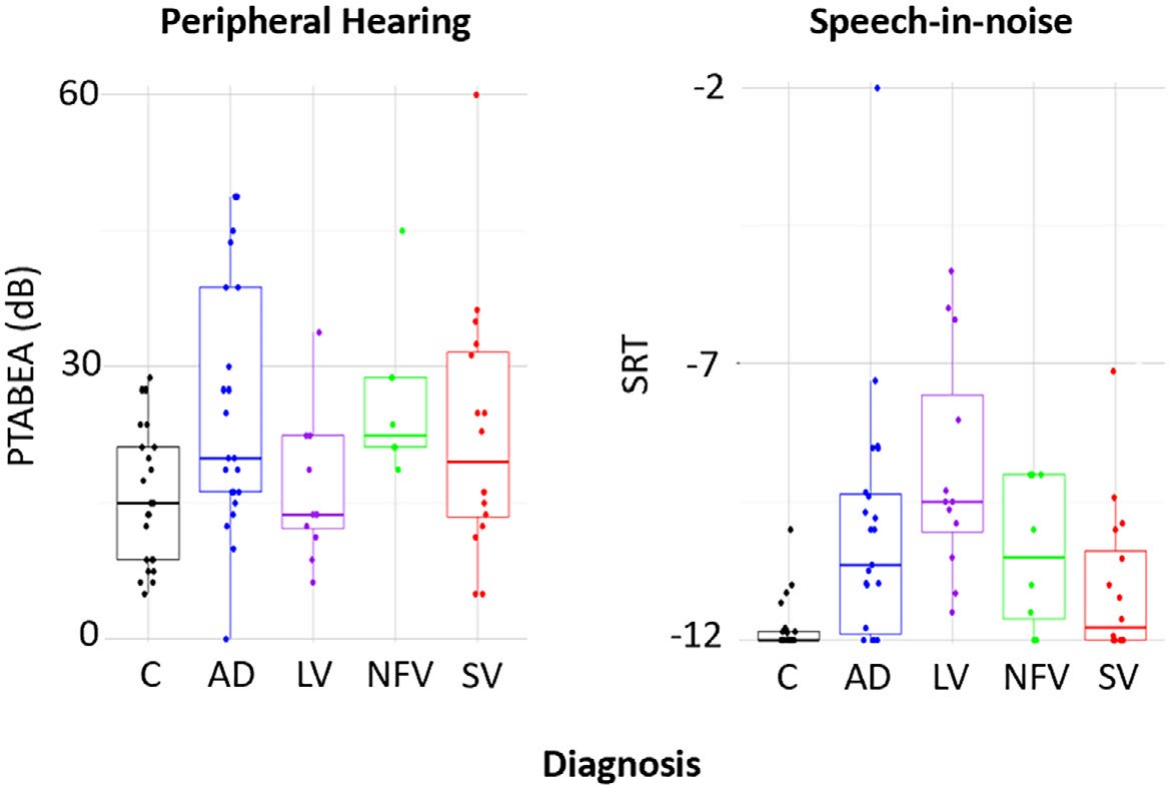
Peripheral hearing and speech‐in‐noise perception in each participant group. Box‐and‐whisker plots illustrate scores on pure tone audiometry (better ear average, PTABEA; left panel) and speech‐in‐noise perception (indexed using 50% speech reception threshold, SRT; right panel), for all individuals in each participant group; a higher score indicates a poorer hearing performance. Boxes code interquartile range; whiskers code 95% confidence intervals; central horizontal lines code median scores; dots show individual participant scores. See Table 1 for quantitative mean group comparisons. AD, patient group with typical Alzheimer's disease; C, cognitively‐healthy control group; LV, patient group with logopenic variant primary progressive aphasia; NFV, patient group with nonfluent/agrammatic variant primary progressive aphasia; SV, patient group with semantic variant primary progressive aphasia.

General demographic and neuropsychological performance data by group are presented in Table 1. Peripheral and speech‐in‐noise characteristics by group are presented in Table 1 and Figure 1.

Participant groups differed significantly in mean age (*F*(4,78) = 3.923, *p* = 0.006; patients with AD and nfvPPA being on average older and patients with svPPA younger than controls), sex distribution (*p* = 0.016; females under‐represented in the lvPPA group), and handedness (*p* = 0.017; left handedness over‐represented in the svPPA group). As anticipated, patient groups all performed significantly worse than cognitively healthy older individuals in the Mini‐Mental State Examination (MMSE) (*X* ^2^ (4) = 42.324, *p* < 0.001), and the lvPPA group performed significantly worse than the nfvPPA group.

The regression model showed a significant effect of diagnostic group on PTABEA (*F*(4,72) = 4.170, *p* = 0.004, η2 = 0.14, 95% confidence interval (CI) [0.01, 1.00]); post hoc comparisons showed that the AD (*p* = 0.005) and nfvPPA (*p* = 0.037) groups had significantly poorer peripheral hearing score than cognitively‐healthy controls. For SRT, there was also a significant effect of diagnostic group (*F*(4,72) = 9.080, *p* < 0.001, η2 = 0.34, CI [0.17, 1.00]). Compared to cognitively‐healthy older individuals, the AD (*p* = 0.001), lvPPA (*p* < 0.001) and nfvPPA (*p* = 0.044) groups all had significantly poorer SRT scores. Comparing syndromic groups, the lvPPA group had significantly poorer SRT scores than the svPPA group (*p <* 0.001), nfvPPA group (*p* = 0.037), and AD group (*p* = 0.042). Parallel non‐parametric and reduced analyses excluding those participants performing at ceiling on the digit‐triplet test and an AD outlier with SRT −2 yielded substantially similar findings (details in Supporting Information).

**FIGURE 2:**
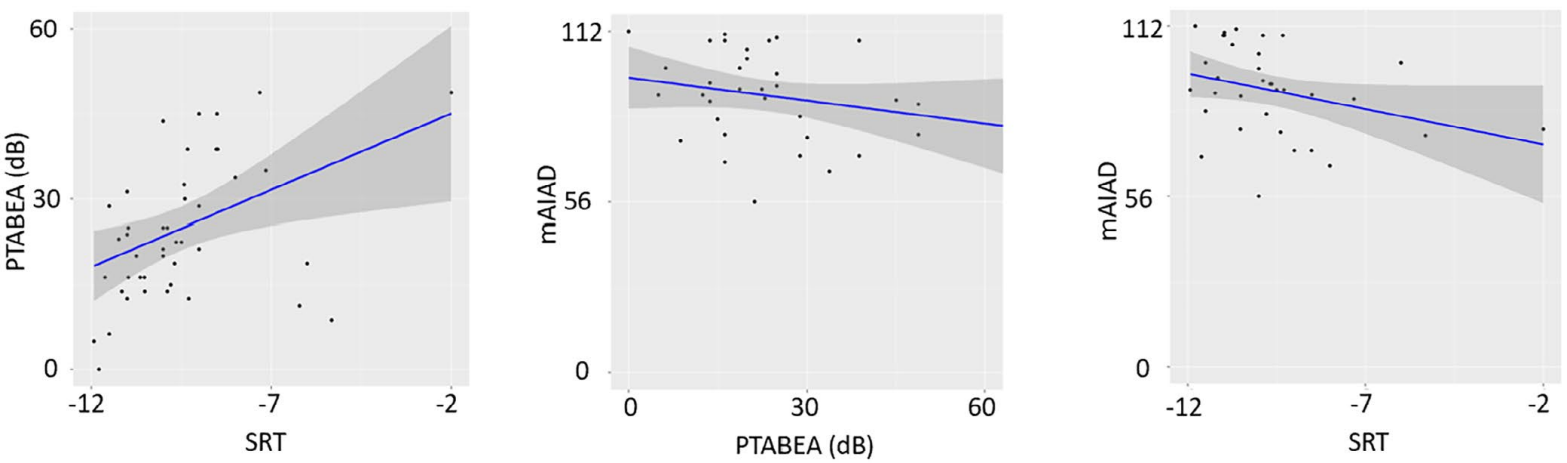
Significant functional correlations of speech‐in‐noise perception in patients. The figure shows significant correlations between speech‐in‐noise perception (50% speech reception threshold, SRT; see text), peripheral hearing score (pure tone audiometry better ear average, PTABEA) and daily life hearing and communication function (modified Amsterdam Inventory for Auditory Disability and Handicap, mAIAD) in the combined patient cohort. Grey envelopes signify 95% confidence intervals.

In the subcohort with ceiling‐performance participants removed, PTABEA was positively associated with age and SRT across all participants (both *p* < 0.001), and with SRT in combined patient groups (*p* = 0.002) and in the AD group alone (*p* < 0.002). SRT was not significantly associated with reverse max digit span (*p =* 0.053) in the patient cohort. mAIAD total score showed no significant association with PTABEA (*p =* 0.123) but was significantly negatively associated with SRT (*p =* 0.027) (Figure 2; see Table S1 for all correlation statistics).

**FIGURE 3:**
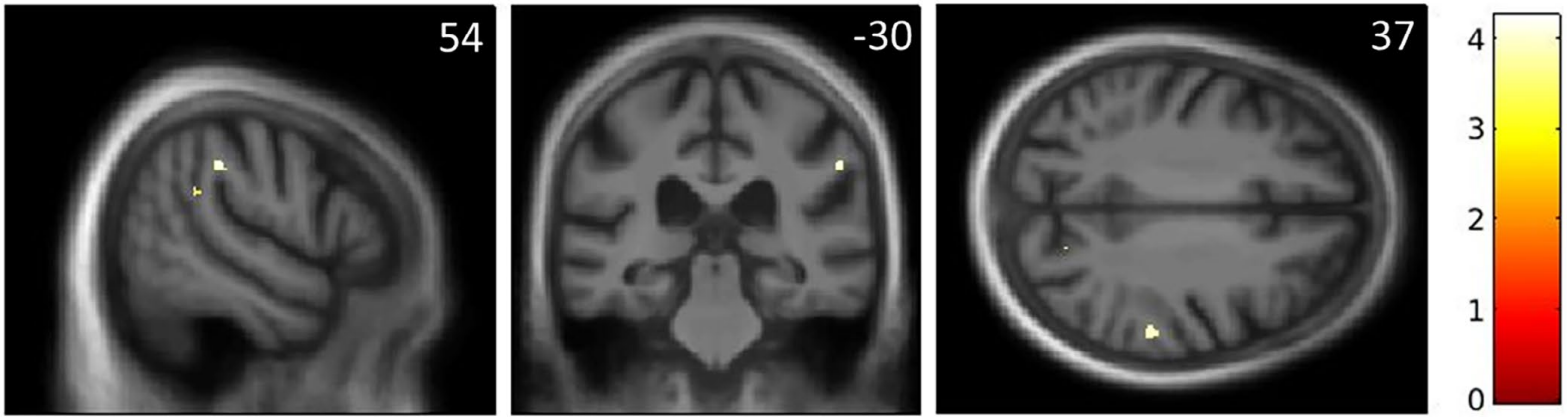
Regional grey matter atrophy associated with impaired speech‐in‐noise perception. The figure shows a statistical parametric map of voxels where grey matter atrophy was significantly associated with elevated speech reception threshold in a voxel‐based morphometric analysis of the combined patient cohort. Statistical parametric maps were generated using an initial cluster‐defining threshold (*p* < 0.001) and assessed at peak‐level significance threshold (*p* < 0.05); grey matter atrophy in the right anterior supramarginal gyrus was significantly associated with impaired speech‐in‐noise processing after family‐wise error correction within the pre‐specified neuroanatomical region of interest (see text). The map has been rendered on sagittal (left), coronal (middle) and axial (right) sections of the normalised group mean T1‐weighted structural MR brain image in MNI space; coordinates of each section plane (mm) are indicated. The colour bar (right) codes voxel‐wise *t*‐values.

Across the combined patient cohort, increased (worse) SRT was significantly associated with regional grey matter atrophy in the right anterior supramarginal gyrus (*p* = 0.024; peak voxel MNI coordinates *x* = 54, *y* = −30, *z* = 37), at threshold *p* < 0.05_FWE_ within the relevant prespecified neuroanatomical region of interest (Figure 3). No other significant grey matter associations of SRT and no significant grey matter correlates of PTABEA were identified at the prescribed threshold (all *p* > 0.05).

## 4 Discussion

Here we have shown that patients with AD and two canonical PPA syndromes, nfvPPA and lvPPA, have impaired speech‐in‐noise perception (indexed by SRT) relative to cognitively‐well older individuals. SRT was correlated with peripheral hearing function; however, syndromic impairments in SRT were evident after taking into account both peripheral hearing performance and potentially confounding general cognitive and executive indices: taken together, these findings suggest that SRT indexes a separable auditory brain process that is vulnerable in dementia syndromes. Moreover, SRT—unlike PTA—predicted real‐world hearing symptoms (as indexed using mAIAD score) in these dementia syndromes. Dementia syndromes were stratified by their SRT profiles: speech‐in‐noise perception was more impaired in lvPPA than other syndromes, but unaffected in svPPA. Our findings build on emerging evidence for brain hearing deficits in AD and PPA [4, 6, 8, 9, 10, 11, 12, 13, 14, 18, 19, 20, 21, 22, 23, 24, 29]. In line with this interpretation, a structural neuroanatomical correlate of speech‐in‐noise perception across the patient cohort was identified in the right anterior supramarginal gyrus. In addition, peripheral hearing acuity (as indexed by PTABEA) was impaired in AD and nfvPPA but not in other PPA syndromes, relative to cognitively‐well older individuals and after adjusting for age.

Difficulty parsing the auditory environment is a feature of AD and its variant syndromes [6, 14, 22], and may disrupt daily‐life communication from an early stage of the illness, especially in lvPPA [10]; this difficulty is also frequent early in the course of nfvPPA [9]. Speech signals have highly‐salient status in many everyday listening scenarios, which often require parsing of speech from background noise: impaired processing of acoustically‐degraded speech has been demonstrated in AD, lvPPA, and nfvPPA [23], and the present work shows that this impairment importantly extends to the perception of speech embedded in background noise. Although speech‐in‐noise perception is affected by peripheral hearing loss, these findings demonstrate that central processes determine the performance profiles that characterise dementia syndromes, and further suggest that these processes are specifically auditory in nature, rather than attributable to more general cognitive deficits. lvPPA may constitute an auditory ‘extreme phenotype’ within the AD spectrum: the more severe impairment of speech‐in‐noise perception in this syndrome may signify synergistic deficits impacting auditory scene parsing, auditory working memory and phoneme transcoding [19, 30, 31]. While nfvPPA is also associated with impaired phonemic coding, this is in the context of a broader impairment of auditory feature analysis [29, 31]. In addition, patients with nfvPPA here had impaired PTA performance, in line with previous evidence in this syndrome [11] and likely due to top‐down dysregulation of auditory efferent pathways modulating signal transmission, and sensitivity of acoustic target detection in the peripheral hearing apparatus [32]. Although we did not specifically predict a PTA deficit in our AD group, PTA performance in AD is variable and potentially likewise affected by top‐down cognitive factors that remain ill‐defined [33, 34]. Intact speech‐in‐noise perception in the svPPA group here is in line with previous evidence for sparing of auditory scene analysis and perceptual decoding in this syndrome [6, 14].

The linking of impaired speech‐in‐noise perception to atrophy of the right supramarginal gyrus accords with previous evidence implicating the inferior parietal cortex in auditory scene analysis in the healthy brain [35, 36] and in the pathophysiology of impaired auditory scene analysis in AD syndromes, including deficits of spatial hearing and the ‘cocktail party effect’ that normally allows tracking of speech over background babble [18, 22]. This region is a key component of the temporo‐parietal ‘default mode’ network, a core target of AD pathology that has been linked to the pathogenesis of a range of central hearing abnormalities across the AD clinical spectrum [17]. Auditory dysfunction in nfvPPA has also been linked to temporo‐parietal mechanisms [17, 19]. The relative vulnerability of these regions, coupled with the heavy computational demands of speech‐in‐noise perception and related central auditory processes, may account for the early development of hearing changes in AD, lvPPA and nfvPPA [9, 10].

From a clinical perspective, our findings support the clinical impression that people with AD and PPA often have difficulty following speech in noisy environments, and suggest that this may be a particularly salient limitation in lvPPA versus other dementia syndromes. Further, this study corroborates previous evidence [34, 37, 38] that tests of brain hearing function such as speech‐in‐noise perception might be harnessed to predict everyday hearing and communication abilities, outperforming PTA for this purpose. In people with suspected AD or PPA who have hearing symptoms, speech‐in‐noise perception is likely to add diagnostic value over standard clinical PTA, and particularly in light of its demonstrated neural substrate, warrants further investigation as a potential physiological biomarker of early clinical AD [24], lvPPA and nfvPPA [9, 10]. This is an issue of increasing importance with the advent of widely accessible, blood‐based markers of AD pathology and the first disease modifying treatments [39]. Beyond diagnosis, testing speech‐in‐noise perception could potentially serve as a marker for the efficacy of hearing devices and neurorehabilitative interventions and indeed, help guide the development of such interventions. An important recommendation for clinicians from this work is to assess both PTA and speech‐in‐noise perception in characterising real‐world hearing in people presenting with dementia, and to consider the likely impact of background noise on everyday communication (motivating trials of dementia‐friendly augmentation aids and strategies such as subtitles and quiet spaces). This might be especially apposite in PPA, where the prominence of language output issues can readily obscure listening difficulties and management needs [9, 10].

This study has several limitations that should direct future work. The patient cohort was relatively small; while a post hoc power analysis suggested that our SRT model was well powered (0.99) to detect the effect of diagnostic group and the associated effect size was large (η2 = 0.34), it will be essential in future work to extend and replicate our findings in larger and more diverse patient cohorts. Ideally, future studies should be longitudinal, to define the time course of hearing changes in relation to other, standardised disease biomarkers and assess the potential of speech‐in‐noise perception for tracking disease evolution [34]. Particularly pertinent to PPA syndromes, it will be important to engage multi‐centre (multi‐lingual) collaborations, to assess the extent to which brain hearing test performance is modulated by native language [40]. Functional neuroimaging techniques that can capture connectivity changes between cortical and subcortical auditory structures will be required to define the pathophysiological mechanisms of central hearing deficits, in particular the basis for reduced PTA performance in nfvPPA. Studying other central hearing tests alongside speech‐in‐noise perception is likely to inform diagnostic characterisation and intervention planning: these tests might potentially overcome the ceiling effects evident in the present SRT data (see Figure 1), and optimally target particular dementia syndromes [37]. For example, patients with svPPA may exhibit altered speech perception on tests manipulating semantic predictability [15, 16]; future work could profitably compare performance across tasks with varying cognitive and linguistic demands, to further disambiguate perceptual and higher‐order effects on brain hearing tests. Here we used an in‐house digit triplet test based on the version used in the UK Biobank study; however, idiosyncrasies in test development preclude direct comparisons across studies using different speech‐in‐noise tests. Finally, more work is needed to explore how deficits on hearing tests relate to hearing difficulties in everyday life in a broader range of dementia syndromes and beyond the laboratory, to assess their functional relevance more fully. This will be essential for designing and evaluating rational, effective interventions to improve daily‐life communication in people living with different forms of dementia.

## Author Contributions

**Benjamin A. Levett:** investigation. **Tim Green:** writing – review and editing. **Nehzat Koohi:** writing – review and editing. **Lucy B. Core:** investigation, writing – review and editing. **Jason D. Warren:** supervision, writing – review and editing, writing – original draft, funding acquisition, visualization, conceptualization, project administration, resources. **Doris‐Eva Bamiou:** writing – review and editing. **Chris J. D. Hardy:** investigation, funding acquisition, writing – review and editing, methodology, writing – original draft, supervision, visualization, conceptualization, software, project administration, validation, resources. **Charles R. Marshall:** writing – review and editing. **Madison Tutton:** writing – review and editing. **Jessica Jiang:** investigation. **Sophie A. Froud:** writing – review and editing, formal analysis, writing – original draft, visualization, data curation, validation, methodology.

## Funding

The Dementia Research Centre is supported by Alzheimer's Research UK, Brain Research Trust and The Wolfson Foundation. This work was supported by the Alzheimer's Society (Grant AS‐PG‐16‐007 to J.D.W.), the Royal National Institute for Deaf People (G105 to J.D.W.), Alzheimer's Research UK, and the National Institute for Health and Care Research University College London Hospitals Biomedical Research Centre (NIHR204280). S.A.F. is supported by a Frontotemporal Dementia Research Studentship in Memory of David Blechner (funded through The National Brain Appeal). B.A.L. is supported by the Alzheimer's Society. J.J. was supported by a Frontotemporal Dementia Research Studentship in Memory of David Blechner (funded through The National Brain Appeal). L.B.C. was supported by a UCL Research Excellence Scholarship. N.K. is supported by a fellowship grant from the National Institute for Health Research (HEE/NIHR ICA Programme Clinical Lectureship NIHR302201). D.E.B. is supported by the Royal National Institute for Deaf People. C.R.M. is supported by a grant from Bart's Charity and the National Institute for Health and Care Research (NIHR204280). C.J.D.H. was supported by an RNID‐Dunhill Medical Trust Pauline Ashley Fellowship (Grant PA23_Hardy), a Fellowship award from Alzheimer's Society, UK (Grant 627) and the National Institute for Health and Care Research (NIHR204280).

## Conflicts of Interest

The authors declare no conflicts of interest.

## Supporting information


**Supporting Information:** ene70701‐sup‐0001‐Supporting Information.docx.

## Data Availability

The data that support the findings of this study are available on request from the corresponding author. The data are not publicly available because they contain information that could compromise the privacy of research participants.
