# Supplementary material for: Speech‐In‐Noise Perception in Alzheimer's Disease and Primary Progressive Aphasia
Source: Eur J Neurol. 2026 Jul 14;33(7):e70701. doi: 10.1111/ene.70701 (PMC13367126; doi:10.1111/ene.70701)
Supplement: Supplementary file 1 — Supporting Information: ene70701‐sup‐0001‐Supporting Information.docx. [file ENE-33-e70701-s001.docx]

**Supplementary material. Speech-in-noise perception in Alzheimer’s disease and primary progressive aphasia, by SA Froud et al**

# **Assessment of peripheral hearing**

Peripheral hearing was measured using pure-tone audiometry, which was administered following British Society of Audiology guidelines^1^ at 500, 1000, 2000 and 4000 Hertz (Hz) using either a dual-channel GSI Audiostar Pro audiometer or an Amplivox Screening audiometer model 116 with calibrated, noise-reducing headphones in a quiet room. An average pure-tone threshold was calculated separately for left and right ears of each participant by taking an average hearing level (in decibels, dB) for tone detection across all frequencies.

# **Assessment of speech-in-noise perception**

## **Stimulus synthesis**

The digit triplet test employed here was based on the speech-in-noise task used in the UK Biobank Study^2^ (https://biobank.ndph.ox.ac.uk/showcase/showcase/docs/Hearing.pdf). Key adaptations were the use of a different (young adult male, standard Southern English) speaker, with inclusion of the bisyllabic digit ‘seven’: this ‘seven’ was adopted due to the availability of pre-recorded numbers for our stimulus set, but would also facilitate future translation into languages other than English. Recorded digits were normalised to a fixed intensity level (63 dB) using a Praat script written by Matthew Winn:

(<https://raw.githubusercontent.com/ListenLab/Praat/master/Scale_intensity_all_sounds_in_folder_v1.txt>)

Digits were grouped into triplet sequences using a MATLAB script developed by C.J.D.H. A total of 504 (9 * 8 * 7) unique triplets were generated, with no digits repeated within a triplet and digits within triplets flanked by 100 millisecond silent intervals. To create speech-weighted (‘speech-shaped’) noise, we applied a filter function based on the long-term average spectrum of the digit sequences^3^ to white noise. A Praat script by Daniel McCloy was used to mix the digit triplets with the speech-shaped noise at prescribed signal-to-noise ratios (SNRs) ranging from –12 dB to +8 dB, consistent with the UK Biobank protocol. These ratios are consistent with the noise characteristics of commonly encountered everyday listening environments, such as a railway station or restaurant^4-6^. Ratios were created by varying the noise level over a fixed speech level.

## **Experimental protocol**

The experiment was conducted in a quiet room in UCL Dementia Research Centre. Auditory stimuli were presented to participants binaurally via Audio-Technica ATH-M50X headphones at a comfortable fixed listening level (at least 70 dB). Before the task, participants were told that they would hear some numbers and that their job is to repeat them. Responses were recorded verbatim by the experimenter; no feedback was given about performance. The SNR of each digit triplet was adjusted from trial to trial based on task performance following an adaptive psychophysical staircase protocol, such that a correct (incorrect) score led to an increased (decreased) noise level on the next trial. Fifteen trials were administered to every participant. The first trial was always at 2dB, and the adaptive staircase occurred between trials 2 and 15; the inter-trial noise level step was 4 dB for the first five trials and 2 dB on the remaining trials, finishing on trial 15 with a final speech-to-noise ratio for the individual. If necessary, task instructions were repeated, and breaks were given; participants were allowed to wear hearing aids during testing if prescribed these.

## **Calculation of SRT**

An individual 50% speech intelligibility or speech reception threshold (SRT) was calculated for each participant. For each SNR assessed, each trial was scored according to the number of heard digits repeated (in any order) and scores were summed across trials to give a ‘proportion correct’ score for that SNR. Disregarding order was intended to ensure we were measuring hearing ability (perception of the digits) rather than working memory (sequencing of digits), as working memory impairment might potentially impact sequence ordering in the patients studied here.

The SNR at which >50% of digits were first repeated correctly (SNR_hi_) and the SNR at which just < 50% of digits were repeated correctly (SNR_lo_) were determined together with the percentage proportion of correct digits at each of these SNR levels (%), and the following interpolation formula was applied to derive the SRT (the SNR at which 50% of digits would be intelligible (repeated correctly) by that individual):

*SNR = SNR_lo_ + ( ((50 - %(SNR_lo_) * (SNR_hi_ - SNR_lo_)) / (%(SNR_hi_) - %(SNR_lo_)) )*

The SRT corresponds to the SNR at which small changes in noise level most affect the accuracy of the individual’s speech perception.

# **Brain image acquisition and pre-processing**

The VBM analysis was based on scans from 40 patients (12 AD, 10 lvPPA, 6 nfvPPA, 12 svPPA); excluded were one nfvPPA patient who did not have a brain scan, one AD patient with significant movement artefacts after pre-processing, and one AD and one lvPPA patient with missing WASIMR data.

Volumetric structural brain MRI scans were acquired on a 3Tesla Siemens Prisma scanner, using a 32-channel phased array head coil and following a T1-weighted sagittal 3D magnetization prepared rapid gradient echo (MPRAGE) sequence (echo time = 2.9 ms, inversion time = 900 ms, repetition time = 2200 ms), with dimensions 256 mm × 256 mm × 208 mm and voxel size 1.1 mm × 1.1 mm × 1.1 mm).

Brain images were pre-processed and normalised to standard MNI stereotactic space using SPMv12 (http://www.fil.ion.ucl.ac.uk/spm/software/spm12/) and the DARTEL toolbox with default parameters, running under MATLAB R2014b. Images were smoothed using a 6-mm full-width at half-maximum Gaussian kernel. To control for individual differences in total brain size, total intracranial volume was calculated for each participant by summing white matter, grey matter and CSF volumes post-segmentation.^7^ An explicit brain mask was created using an automatic mask-creation strategy designed previously.^8^ The model did not include grand mean scaling or global normalisation. The PTA model had 35 degrees of freedom and the SRT model 34. In both models, multicollinearity was below 0.4 between all regressors aside from between age and PTABEA, which was 0.55. A study-specific mean brain template image upon which to overlay statistical parametric maps was created by warping all patient’s native-space whole-brain images to the fine DARTEL template and using the ImCalc function to generate an average of these images.

Brain volumes for region of interest analyses correcting for multiple voxel-wise comparisons were derived from a published atlas.^9^ Neuroanatomical regions are shown in Figure S1.

# **Parallel analyses**

To address potential concern over ceiling effects on our SRT results, three additional, parallel analyses were conducted to assess whether the results were altered when assessed non-parametrically, or after excluding ceiling participants and outliers. The non-parametric Kruskal-Wallis ANOVA with SRT and diagnostic group revealed a significant effect of group on SRT score (*X²(*4) = 14.977, *P* =0.005). In the ceiling-excluded participant cohort, the main effect of group remained significant (*X²(*4) = 14.977, *P* = 0.005), with the AD group (*P*= 0.008) and lvPPA group (*P*= 0.017) performing significantly worse than controls. These effects remained significant in the ceiling-excluded participant cohort with the SRT outlier removed, too; *X²(*4) = 18.801, *P* < 0.001, with the AD group (*P*= 0.010) and lvPPA group (*P*= 0.017) performing significantly worse than controls.

# **Supplementary references**

1. British Society of Audiology, 2018. Recommended procedure: pure-tone air-conduction and bone conduction threshold audiometry with and without masking.
2. Stevenson JS, Clifton L, Kuzma E, Littlejohns TJ. Speech-in-noise hearing impairment is associated with an increased risk of incident dementia in 82,039 UK Biobank participants. Alzheimers & Dementia 2021; 18(3): 445–456. doi:10.1002/alz.12416.
3. Quene H, Van Delft LE. Non-native durational patterns decrease speech intelligibility. Speech Communication 2010; 52(11), 911-918. doi:10.1016/j.specom.2010.03.005
4. Singh T, Biggs T, Crossley E, Faoury M, Mahmood, A et al. Noise Exposure on the London Underground, an Observational Study over a Decade. The Laryngoscope 2012; 130: 2891-2895. doi: 10.1002/lary.28547
5. Barlow C, Castilla-Sanchez, F. Occupational noise exposure and regulatory adherence in music venues in the United Kingdom. Noise and Health 2012; 14(57): 86-90. doi*:*10.4103/1463-1741.95137
6. Rusnock CF, Bush PM. Case Study: An Evaluation of Restaurant Noise Levels and Contributing Factors. Journal of Occupational and Environmental Hygiene 2012; 9(6): D108–D113. Doi: 10.1080/15459624.2012.683716
7. Malone IB, Leung KK, Clegg S, Barnes J, Whitwell JL, Ashburner J, Fox NC, Ridgway GR. Accurate automatic estimation of total intracranial volume: A nuisance variable with less nuisance. NeuroImage 2015; 104: 366–372.

https://doi.org/10.1016/j.neuroimage.2014.09.034

1. Ridgway GR, Omar R, Ourselin S, Hill DLG, Warren JD, Fox NC. Issues with threshold masking in voxel-based morphometry of atrophied brains. NeuroImage 2009; 44(1): 99–111. https://doi.org/10.1016/j.neuroimage.2008.08.045
2. Desikan RS, Ségonne F, Fischl B, Quinn BT, Dickerson BC, Blacker D, Buckner RL, Dale AM, Maguire RP, Hyman BT, Albert MS, Killiany RJ. An automated labeling system for subdividing the human cerebral cortex on MRI scans into gyral based regions of interest. Neuroimage 2006; 31(3): 968–980. https://doi.org/10.1016/j.neuroimage.2006.01.021

# **Table S1. Correlations between variables of interest**

| **Correlation** | **Participants analysed** | **Statistic** |
| --- | --- | --- |
| **Ceiling-excluded cohort (see text)** | | |
| **PTABEA and SRT** | All participants | ***r*(50) = 0.500, *P <*0.001** |
| **PTABEA and age** | All participants | ***r*(50) = 0.581, *P <*0.001** |
| **PTABEA and SRT** | All patients | ***r*(42) = 0.446, *P =* 0.002** |
| SRT and DS reverse | All patients | *r*(42) = -0.316, *P =* 0.053 |
| **SRT and mAIAD** | All patients | ***r*(34) = -0.380, *P=* 0.027** |
| PTABEA and mAIAD | All patients | *r*(34) = -0.270, *P=*0.123 |
| **PTABEA and SRT** | AD patients | ***r*(15) = 0.819, *P* <0.001** |
| PTABEA and DS reverse | AD patients | *r*(15) = -0.244, *P* *=*0.382 |
| SRT and DS reverse | AD patients | *r*(15) = -0.347, *P* *=*0.206 |
| **SRT and mAIAD** | AD patients | ***r*(12) = -0.754, *P* *=*0.002** |
| **PTABEA and mAIAD** | AD patients | ***r*(12) = -0.544, *P* *=*0.044** |
| **Removal of one SRT outlier (see text)** | | |
| **PTABEA and SRT** | All participants | ***r*(49) = 0.406, *P* <0.001** |
| **PTABEA and age** | All participants | ***r*(49) = 0.560, *P* <0.001** |
| **PTABEA and SRT** | All patients | ***r*(41) = 0.335, *P =* 0.012** |
| SRT and DS reverse | All patients | *r*(41) = -0.264, *P =* 0.116 |
| **SRT and mAIAD** | All patients | ***r*(33) = -0.746, *P =* 0.045** |
| PTABEA and mAIAD | All patients | *r*(33) = -0.240, *P =* 0.179 |
| **PTABEA and SRT** | AD patients | ***r*(14) = 0.784, *P* <0.001** |
| PTABEA and DS reverse | AD patients | *r*(14) = -0.094, *P* =0.750 |
| SRT and DS reverse | AD patients | *r*(14) = -0.214, *P* *=*0.462 |
| **SRT and mAIAD** | AD patients | ***r*(11) = -0.737, *P* *=*0.004** |
| PTABEA and mAIAD | AD patients | *r*(11) = -0.481, *P* *=*0.100 |

These correlation analyses were run removing individuals who performed at SRT ceiling level; this excluded 67% of the healthy control group, 26% of the Alzheimer’s disease group, 0% of the lvPPA group, 25% of the nfvPPA group, and 44% of the svPPA group. All significant results in bold correspond to Spearman correlation *P* values <0.05. DS, max digit span; mAIAD, modified Amsterdam Inventory for Auditory Disability and Handicap; PTABEA, pure tone audiometry better ear average; SRT, 50% speech reception threshold (see text).

**Figure S1. Representative sections of neuroanatomical regions that were used for multiple voxel-wise comparisons correction in region-of-interest analyses.**

**
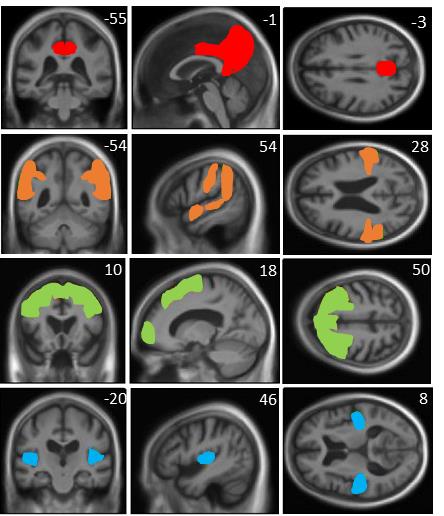
**

Regions of interest used for small volume correction in the voxel-based morphometry analyses (based on pre-specified hypotheses) are overlaid on coronal (left), sagittal (middle) and axial (right) sections of the group mean structural brain image. Red overlays correspond to posteromedial regions including bilateral posterior cingulate and precuneus; orange to lateral temporoparietal regions including bilateral superior temporal gyri, angular gyri and anterior and posterior supramarginal gyri; green to dorsolateral prefrontal cortical regions including bilateral frontal pole, middle frontal gyri and superior frontal gyri; blue to Heschl’s gyri.
